# Complete chloroplast genome of *Cerasus kumanoensis* (Rosaceae), a wild flowering cherry endemic to Kii Peninsula, Japan

**DOI:** 10.1080/23802359.2019.1666052

**Published:** 2019-09-16

**Authors:** Zhong-Shuai Sun, Xian-Gui Yi, Xin-Hong Liu, Toshio Katsuki

**Affiliations:** a Zhejiang Provincial Key Laboratory of Plant Evolutionary Ecology and Conservation, Taizhou University, Taizhou, China;; b Co-Innovation Center for the Sustainable Forestry in Southern China, College of Biology and Environment, Nanjing Forestry University, Nanjing, Jiangsu, China;; c Zhejiang Academy of Forestry, Hangzhou, China;; d Tama Forest Science Garden, Forestry and Forest Products Research Institute, Hachioji, Tokyo, Japan

**Keywords:** *Cerasus kumanoensis*;·flowering cherries, chloroplast genome, phylogenomics

## Abstract

*Cerasus kumanoensis* is a recently described wild cherry species from the Kii Peninsula, Japan. Here we determined the first complete chloroplast genome of *C. kumanoensis* using genome skimming approach. The cp genome was 157,898 bp long, with a large single-copy region (LSC) of 85,926 bp and a small single-copy region (SSC) of 19,070 bp separated by a pair of inverted repeats (IRs) of 26,451 bp. It encodes 129 genes, including 84 protein-coding genes, 37 tRNA genes, and 8 ribosomal RNA genes. Besides, we reconstructed the phylogeny of *Prunus* using maximum likelihood (ML) method, including our data and previously reported cp genomes of related taxa. The phylogenetic analysis indicated that *C. kumanoensis* is close related with a group including *Prunus takesimensis* and *P. speciosa*.

*Cerasus* is a flowering tree genus with high ornamental and economic values in Japan (Ohba 2001; Ikeda et al. 2017). According to recent researches (Shi et al. 2013; Chin et al. 2014), genus *Cerasus* or subgenus *Cerasus* in *Prunus* has been shown a monophyletic group distinguished from apricot, peach, plum or bird cherry. In this article, we use genus *Prunus sensu lato* except for *Cerasus kumanoensis* T. Katsuki to prevent confusion. *C. kumanoensis* (Kumano cherry) is a recently described species, from the southern Kii Peninsula, Honshu, Japan. It can be distinguished easily from its related taxa by several morphological and phenological characteristics (Katsuki 2018), but the genetic relationship of *C. kumanoensis* relative to other flowering cherries has not been well established. By taking advantages of next-generation sequencing technologies that efficiently provide the chloroplast (cp) genomic resources of our interested species, we can rapidly access the abundant genetic information for phylogenetic research and conservation genetics (Liu et al. 2017, 2018). Therefore, we sequenced the whole chloroplast genome of *C. kumanoensis* to elucidate its phylogenetic relationship with other flowering cherries.

Total genomic DNA was extracted from silica-dried leaves collected from Kozagawa in southern of Wakayama Pref., Japan using a modified CTAB method (Doyle and Doyle 1987). The voucher specimen (sun1704047) was collected and deposited in the Herbarium of Taizhou University. DNA libraries preparation and pair-end 125 bp read length sequencing were performed on the Illumina HiSeq 2500 platform. About 8.38 Gb of raw data were trimmed and assembled into contigs using CLC Genomics Workbench 8. Then, all the contigs were mapped to the reference cp genome of *Prunus speciosa* (Koidz.) Nakai (MH998233; Sun et al. 2019) using BLAST (NCBI BLAST v2.2.31) search and the draft *cp* genome of *P. speciosa* was constructed by connecting overlapping terminal sequences in Geneious R11 software (Biomatters Ltd., Auckland, New Zealand). Gene annotation was performed via the online program Dual Organellar Genome Annotator (DOGMA; Wyman et al. 2004).

The complete cp genome of *C. kumanoensis* (GenBank accession MN245147) was 157,898 bp long consisting of a pair of inverted repeat regions (IRs with 26,451 bp) divided by two single-copy regions (LSC with 85,926 bp; SSC with 19,070 bp). The overall GC content of the total length, LSC, SSC, and IR regions were 36.7%, 34.6%, 30.3% and 42.5%, respectively. The genome contained a total of 129 genes, including 84 protein-coding genes, 37 tRNA genes and 8 rRNA genes.

To determine the phylogenetic position of newly sequenced *C. kumanoensis*, phylogenetic analysis was conducted along with 18 representative *Prunus* species and two outgroup taxa. We reconstructed a phylogeny employing the GTR + G model and 1000 bootstrap replicates under the maximum-likelihood (ML) inference in RAxML-HPC v.8.2.10 on the CIPRES cluster (Miller et al. 2010). The ML tree (Figure 1) was consistent with the most recent phylogenetic study on *Prunus* (Shi et al. 2013; Chin et al. 2014). *Cerasus kumanoensis* exhibited the closest relationship with *Prunus takesimensis* Nakai. However, the classification systems for a group including P. takesimensis and P. speciosa (Koidz.) Nakai is not established. It is necessary to analyze more samples including P. jamasakura Siebold ex Koidz and P. leveilleana Koehne in order to clarify the accurate phylogenetic relationship.

**Figure 1.**
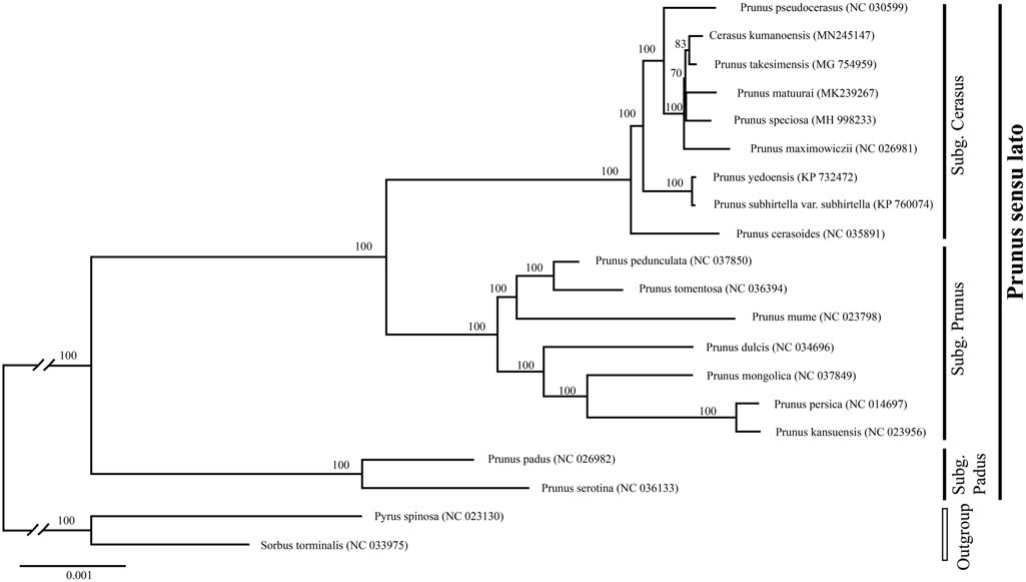
Phylogenetic tree reconstruction of 18 taxa of *Prunus* and two outgroups using ML method. Relative branch lengths are indicated. Numbers near the nodes represent ML bootstrap value. The scientific names of these species are debated.
